# Uterine Preservation in a Case of Isthmic Placenta Accreta With Massive Obstetric Hemorrhage: A Case Report

**DOI:** 10.7759/cureus.109463

**Published:** 2026-05-22

**Authors:** Benabdeslam Rim, Anibri Mouna, Khalid Fathi

**Affiliations:** 1 Department of Obstetrics and Gynecology, Oncology and High-Risk Pregnancy, Souissi Maternity Hospital, Rabat, MAR

**Keywords:** conservative management, isthmic placenta accreta, obstetric hemorrhage, placenta accreta spectrum, uterine preservation

## Abstract

Placenta accreta spectrum (PAS) disorders are associated with significant maternal morbidity, often requiring hysterectomy. Conservative management remains controversial but may be considered in selected cases. We report a case of isthmic placenta accreta in a 32-year-old multiparous woman with a prior cesarean section. The patient presented with antepartum hemorrhage and was diagnosed with placenta previa with suspected accreta on magnetic resonance imaging (MRI). An emergency cesarean section was performed following massive hemorrhage. Partial placental removal was achieved, while the adherent isthmic portion was left in situ and managed conservatively with hemostatic sutures. Despite significant intraoperative bleeding requiring transfusion, the patient had a favorable outcome, with complete spontaneous resorption of retained placental tissue over three months. This case highlights the feasibility of conservative management in selected cases of isthmic placenta accreta.

## Introduction

Placenta accreta spectrum (PAS) disorders are characterized by abnormal trophoblastic invasion into the myometrium and represent one of the leading causes of severe obstetric hemorrhage and maternal morbidity worldwide [1]. The incidence of PAS has risen considerably over recent decades, mainly due to increasing cesarean delivery rates and the growing prevalence of uterine scarring [2,3].

The major risk factors for PAS include placenta previa, previous cesarean section, prior uterine surgery, and multiparity [3]. Among these disorders, isthmic placenta accreta is an uncommon localization that poses significant diagnostic and therapeutic challenges because of its proximity to the cervix, altered vascularization, and high hemorrhagic potential [4].

Prenatal diagnosis relies primarily on ultrasonography, which remains the first-line imaging modality for identifying placental invasion. Magnetic resonance imaging (MRI) may provide complementary information in complex, posterior, or atypical cases and can help assess the depth and topography of invasion [5,6].

The standard treatment for PAS traditionally consists of cesarean hysterectomy without attempting placental removal in order to minimize catastrophic bleeding [7]. However, conservative uterine-preserving approaches, including leaving the placenta partially or completely in situ, have increasingly been reported in selected patients wishing to preserve fertility [8,9]. Despite growing interest in conservative management, evidence regarding its use in rare forms such as isthmic placenta accreta remains limited.

Reporting such cases is important to improve understanding of surgical management strategies, maternal outcomes, and the feasibility of uterine preservation in this insufficiently studied subgroup of PAS disorders. We present a rare case of isthmic placenta accreta complicated by massive obstetric hemorrhage that was successfully managed conservatively with preservation of the uterus.

## Case presentation

Clinical history and presentation

A 32-year-old woman, gravida 3 para 1, with a history of one previous cesarean section performed for suspected macrosomia and one spontaneous miscarriage without curettage, was admitted for vaginal bleeding outside labor at term pregnancy. On admission, the patient was hemodynamically stable. Obstetric examination revealed no uterine contractions.

Investigations

Magnetic resonance imaging (MRI) demonstrated complete placenta previa with abnormal placental invasion localized to the isthmic region over the previous cesarean scar, raising suspicion for isthmic placenta accreta (Figures 1a, 1b). Ultrasound examination revealed a singleton pregnancy with positive fetal cardiac activity in breech presentation and an estimated fetal weight of 4250 g. The placenta was posterior and partially covered the internal cervical os (Figure 2). A few placental lacunae were noted without clear ultrasonographic signs of placenta accreta spectrum disorder. No retroplacental hematoma or intraperitoneal fluid was identified. Cardiotocography (CTG) demonstrated a normal baseline fetal heart rate, moderate baseline variability, presence of accelerations, absence of decelerations, and no uterine activity, consistent with a reassuring tracing according to the National Institute for Health and Care Excellence (NICE) criteria (Figure 3).

**Figure 1:**
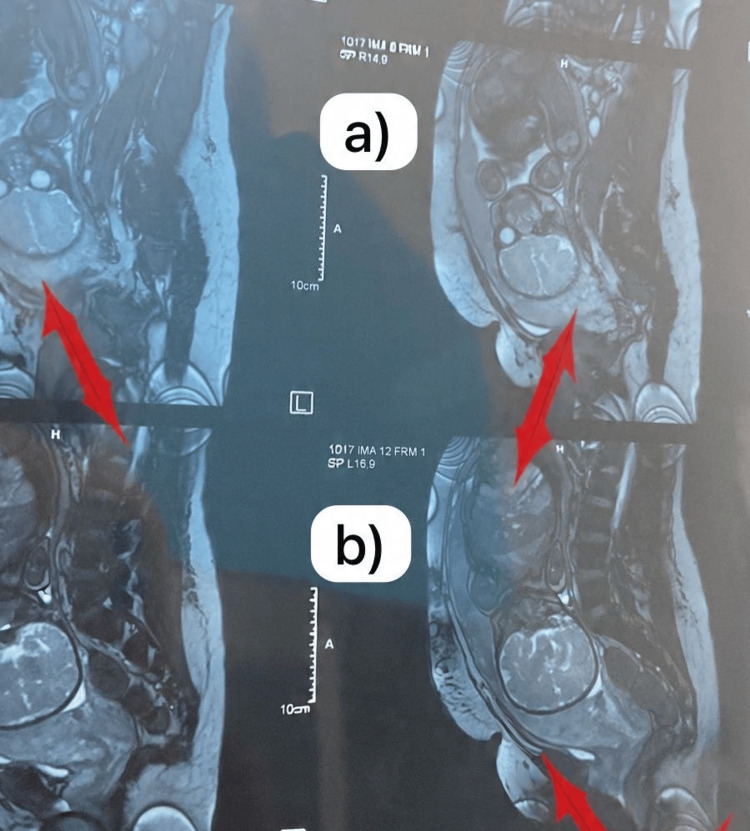
MRI findings of isthmic placenta accreta (a) and (b). (a) Sagittal T2-weighted magnetic resonance image demonstrating complete placenta previa, with the placenta entirely covering the internal cervical os. The placenta appears heterogeneous with areas of abnormal signal intensity. The arrow highlights the region of abnormal placental invasion at the lower uterine segment. (b) Adjacent sagittal section showing placental tissue extending into the isthmic region overlying the prior cesarean scar (arrow). There is loss of the normal hypointense myometrial interface and focal bulging of placental tissue, consistent with placenta accreta spectrum (PAS).

**Figure 2:**
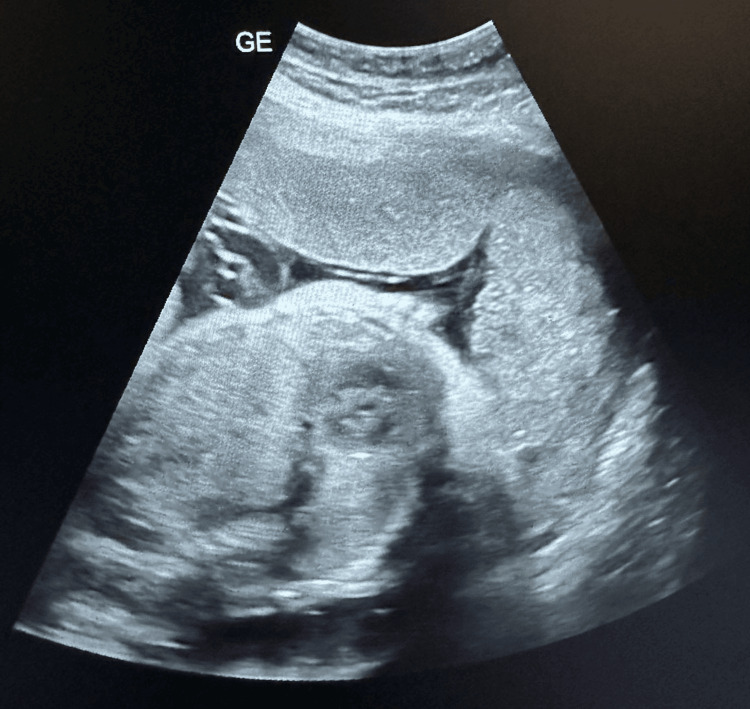
Ultrasound image showing cephalic presentation with posterior placenta partially covering the cervical os without evidence of placenta accreta. Obstetric ultrasound demonstrating a fetus in cephalic presentation (head-down position). The placenta is located on the posterior uterine wall and is noted to be partially covering the internal cervical os, consistent with a low-lying placenta or partial placenta previa. There are no sonographic signs of placental accretization, such as abnormal placental lacunae, loss of the clear zone, or myometrial thinning.

**Figure 3:**
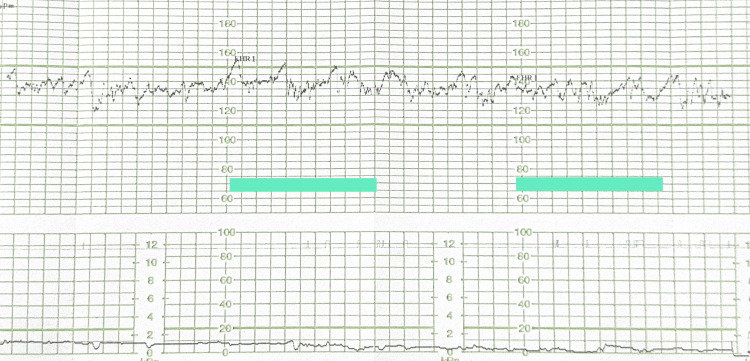
Reactive and normo-oscillatory tracing. Cardiotocography showed a baseline fetal heart rate within the normal range, moderate baseline variability, presence of accelerations, absence of decelerations, and no uterine contractions, consistent with a reassuring/reactive CTG according to NICE guidelines. CTG: cardiotocography; NICE: National Institute for Health and Care Excellence

Treatment

Eight hours after admission, the patient developed a sudden massive vaginal hemorrhage and was transferred emergently to the operating room. A midline supraumbilical laparotomy was performed, followed by a phallic uterine incision. A male neonate in breech presentation weighing 4250 g was delivered successfully.

Approximately 80% of the placenta detached spontaneously, associated with near-total retroplacental hemorrhage. Intraoperatively, a residual placental portion remained firmly adherent to the myometrium and could not be safely separated (Figure 4). The uterus appeared atonic with an apoplectic aspect.

**Figure 4:**
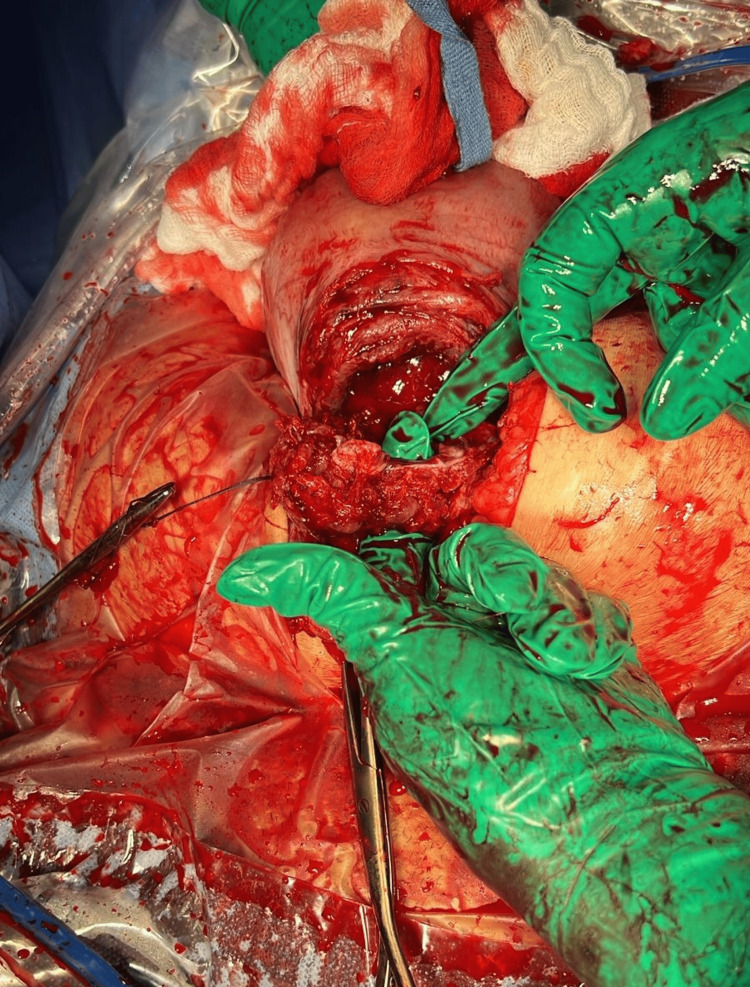
Intraoperative adherent placenta. Intraoperative view showing a non-detachable placenta firmly adherent to the myometrium at the isthmic level, consistent with placenta accreta.

A conservative uterine-preserving surgical approach was adopted. Detached placental tissue was progressively removed using surgical clamps, while the adherent isthmic placental tissue, estimated at 4 cm, was intentionally left in situ (Figure 5). Hemostasis was achieved using X-shaped hemostatic sutures placed over the residual placental tissue (Figure 6).

**Figure 5:**
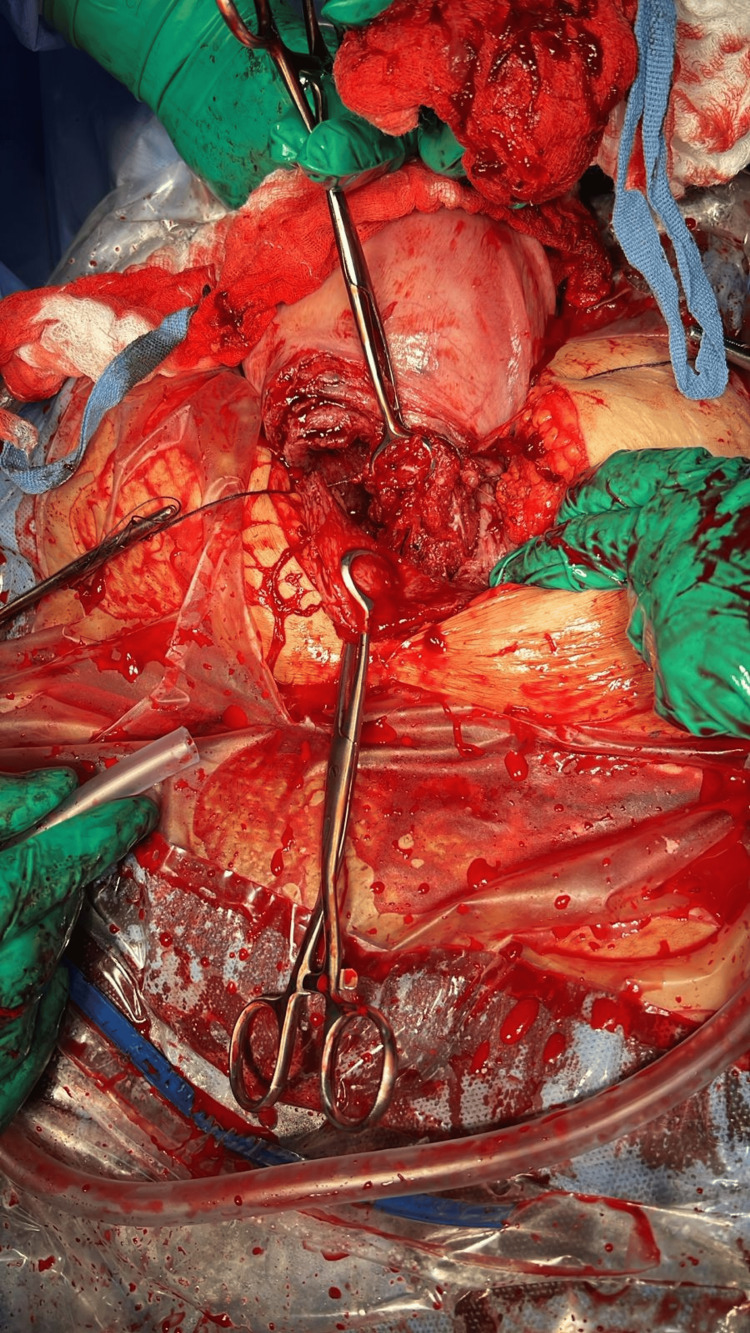
Partial placental resection. Intraoperative partial resection of the detached placental tissue was performed progressively using surgical clamps, avoiding manual removal of the adherent portion.

**Figure 6:**
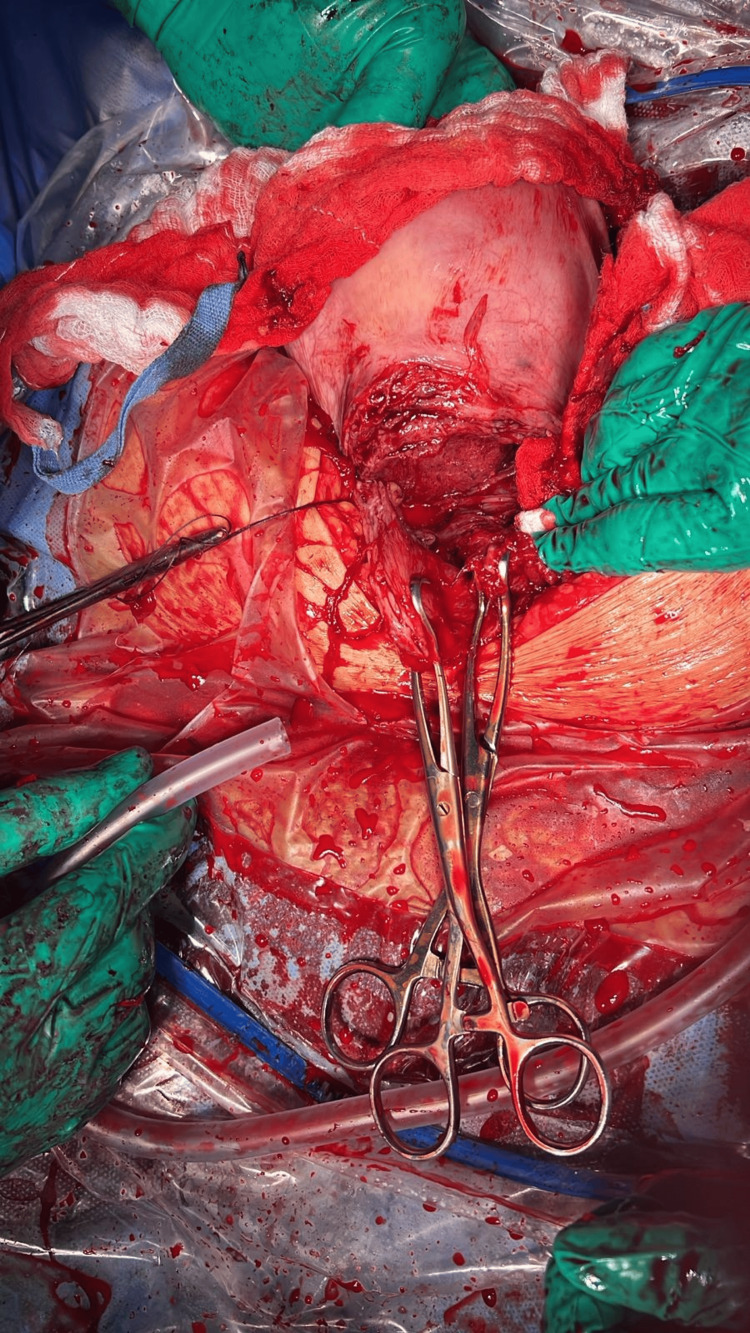
Residual placenta left in situ. Residual placental tissue adherent to the myometrium at the isthmic level is intentionally left in situ after hemostatic suturing to control bleeding and preserve the uterus.

A triple vascular ligation procedure was additionally performed without the administration of oxytocin. Massive transfusion support was required, including five units of packed red blood cells, two units of platelets, and three units of fibrinogen. After hemodynamic stabilization, abdominal lavage was completed, and satisfactory hemostasis was confirmed prior to layered abdominal closure.

Outcome

The postoperative course was favorable. Postoperative ultrasound examination confirmed the presence of a retained placental fragment measuring approximately 4 cm (Figure 7). Despite intermittent episodes of mild bleeding, the patient remained hemodynamically stable and did not require hysterectomy or uterine artery embolization.

**Figure 7:**
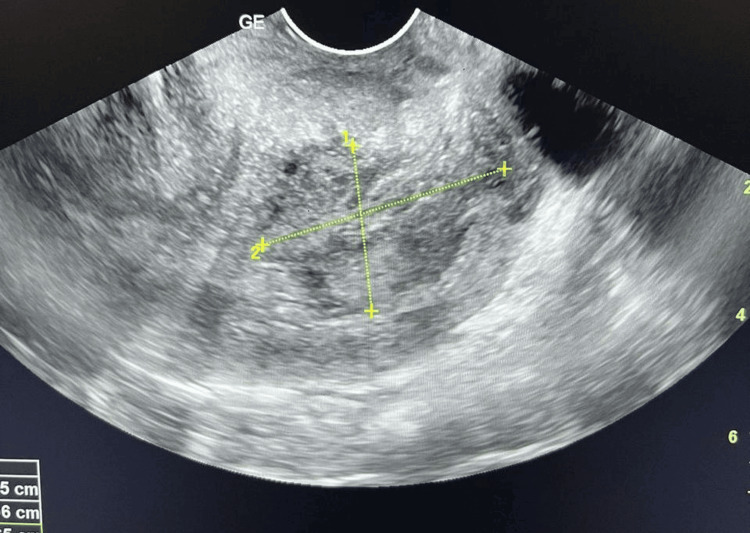
Isthmic retained placental tissue measuring approximately 4 cm in the postpartum period. Transvaginal ultrasound image obtained in the postpartum period demonstrating a heterogeneous intrauterine mass located in the isthmic region. Caliper measurements indicate a lesion of approximately 4 cm in maximal dimension. The echogenic and heterogeneous appearance is consistent with retained placental tissue. This finding is clinically significant due to the associated risks of postpartum hemorrhage and infection, and it may require medical or surgical management depending on the clinical presentation.

Follow-Up

The patient underwent close clinical and ultrasonographic follow-up over a three-month period. Progressive spontaneous resorption of the retained placental tissue was observed, with delayed expulsion of residual placental tissue occurring six weeks postpartum (Figure 8). Complete resolution was achieved without infectious complications or additional surgical intervention, allowing successful uterine preservation.

**Figure 8:**
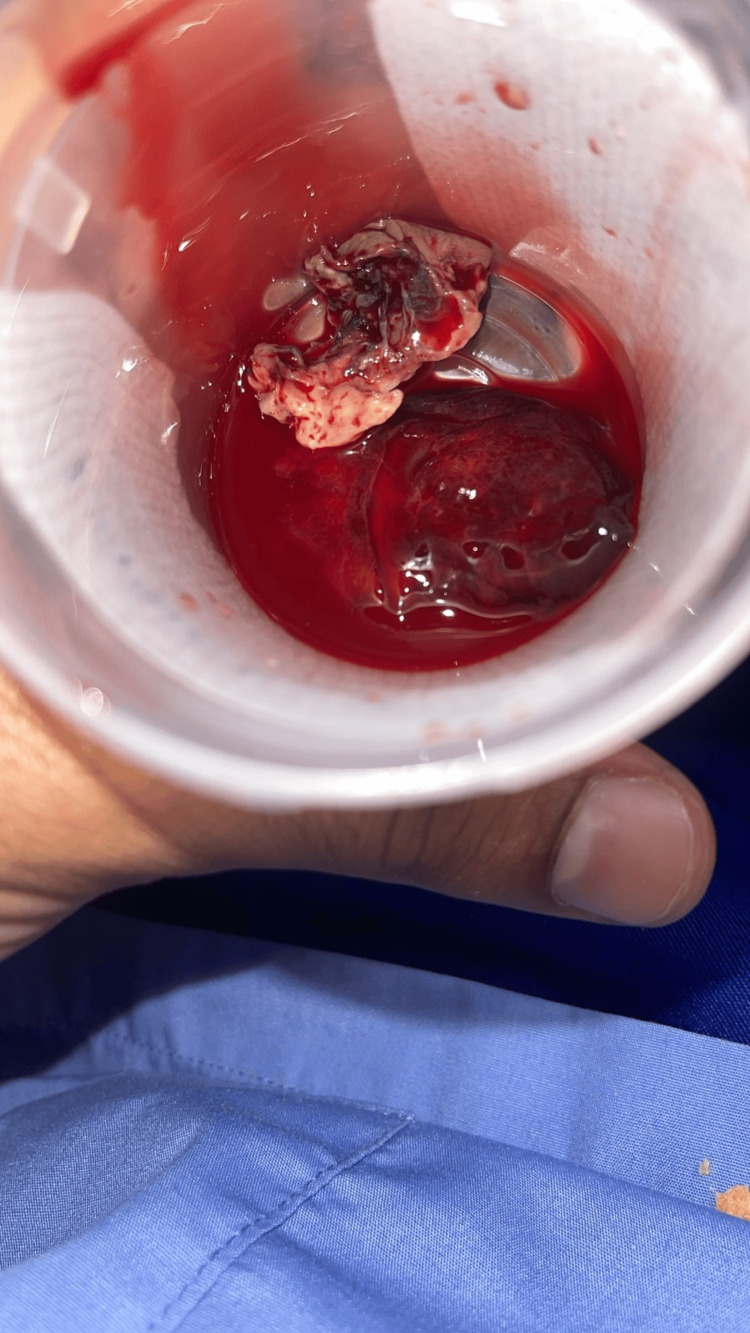
Delayed expulsion of retained placental tissue at six weeks postpartum. Gross image showing the expulsion of blood clots and suspected retained placental tissue occurring approximately six weeks after vaginal delivery. The specimen appears as a heterogeneous mass composed of dark red coagulated blood and pale, irregular fragments consistent with placental remnants.

## Discussion

Placenta accreta spectrum is a major cause of maternal morbidity due to the risk of severe hemorrhage [2,5]. The combination of placenta previa and prior cesarean section significantly increases the risk of PAS, as observed in this case [3]. Isthmic localization is rare and presents specific challenges related to its anatomical position and proximity to the cervix. Diagnosis relies primarily on ultrasound, with MRI providing additional information in complex or atypical cases [7,8].

The standard management of PAS is cesarean hysterectomy without attempting placental removal, as this approach reduces the risk of catastrophic bleeding [5]. However, conservative management strategies, including leaving the placenta in situ, have been increasingly described, particularly in patients desiring fertility preservation [6,9]. In this case, despite a life-threatening hemorrhage, a conservative approach was successfully implemented. Hemostatic suturing of the residual placental tissue allowed effective bleeding control and avoided hysterectomy. The favorable outcome, with spontaneous placental resorption, supports the feasibility of this approach in selected patients. Close follow-up remains essential due to the risk of delayed hemorrhage or infection [9].

## Conclusions

Conservative management with uterine preservation can be a feasible option in carefully selected cases of placenta accreta spectrum, even in the presence of significant hemorrhage. Its success depends on timely decision-making, surgical expertise, and coordinated multidisciplinary care. While not universally applicable, this approach may provide a safe alternative to hysterectomy in stable patients, with the added benefit of preserving future fertility. Careful follow-up remains essential to ensure optimal outcomes. The take-home message was that uterine preservation in isthmic placenta accreta is achievable in carefully selected cases through rapid multidisciplinary intervention, conservative surgical management, and close postoperative follow-up.
